# Anemia and formation of deep vein thrombosis before operation in patients with knee osteoarthritis: a cross-sectional study

**DOI:** 10.1186/s13018-023-03518-w

**Published:** 2023-01-11

**Authors:** Xiaojuan Xiong, Ting Li, Bo Cheng

**Affiliations:** 1 Department of Anesthesiology, Army Medical Center of PLA, Daping Hospital, Army Medical University, 10 ChangjiangZhilu, Yuzhong District, Chongqing, 400042 China; 2 Department of Anesthesiology, The First Affiliated Hospital of Chongqing Medical University Yuzhong District, 1 Youyi Road, Yuzhong District, Chongqing, 400000 China

**Keywords:** Knee osteoarthritis, Deep vein thrombosis, Preoperative, Anemia

## Abstract

**Background:**

Preoperative anemia is a common complication in knee osteoarthritis (KOA) patients. However, the association between anemia and preoperative deep vein thrombosis (DVT) in osteoarthritis patients remains unknown. The aim of this study was to investigate such association.

**Methods:**

In this retrospective study, we included 1005 KOA patients undergoing total knee arthroplasty (TKA) in our hospital. According to preoperative hemoglobin levels, the patients were divided into anemia group and non-anemia group. According to the results of Doppler ultrasonography for the lower extremities, the patients were divided into DVT group and non-DVT group. A logistic model was established through propensity score matching (PSM), with anemia before TKA as the dependent variable, DVT-related variable as the covariate, and 0.03 as the Caliper value. The anemia group and non-anemia group were matched at a 1:1 ratio and 310 successfully matched. After matching, logistic regression analysis was used to evaluate the correlation between preoperative anemia and DVT in KOA patients.

**Results:**

In this study, 342 cases (33.6%) had preoperative anemia and 73 cases (7.2%) had DVT before TKA. After matching, 46 DVT cases (7.42%) were found. By using binary logistic regression after PSM, we found that the risk for preoperative DVT formation in TKA patients with preoperative anemia increased by 1.97 times [95% (CI 1.05–3.69)], *P* = 0.035.

**Conclusion:**

Preoperative anemia is considered as an independent risk factor for the formation of preoperative DVT in KOA patients.

*Trial registration*: ChiCRT2100054844.

## Background

Knee osteoarthritis (KOA) is a chronic degenerative joint disease, and total knee arthroplasty (TKA) is the gold standard treatment for osteoarthritis (OA) [1]. Patients undergoing TKA are at high risk for venous thromboembolism (VTE) \*MERGEFORMAT [2]. Without medication intervention, the incidence of asymptomatic DVT after TKA is 40–85%, and that of fatal pulmonary embolism (PE) is 0.87–1.99% [3]. Currently, common agents for DVT prophylaxis are: anticoagulation therapy based on coagulation cascade, such as heparin and rivaroxaban; antiplatelet therapy, such as aspirin. Although previous studies showed that anemia may be one of risk factors for DVT, it remains unclear what role the red blood cell (RBC) plays in the preoperative DVT formation in OA patients.

Preoperative anemia is a prevalent complication in patients undergoing total joint arthroplasty (TJA), with a high incidence of 44%\*MERGEFORMAT [4]. It has been reported that low hemoglobin (Hb) level is a risk factor for VTE in cancer patients [5]. Anemia is a risk factor for cerebral venous thrombosis [6]. Iron-deficiency anemia was reported as an independent predictive factor for VTE recurrence in patients with unprovoked thrombosis [7]. Meanwhile, anemia was found be an independent risk factor for preoperative DVT in patients with hip fracture [8]. For patients with OA and end-stage rheumatoid arthritis (RA), continuous formation of the inflammation mediator, hemolyzed and damaged RBCs are often accompanied by preoperative chronic anemia, some severe cases may have a shortened lifespan. So far, few studies verify whether preoperative anemia is a high risk factor for DVT formation in chronic arthritis patients.

## Materials and methods

### Inclusion and exclusion criteria

*Inclusion* All patients who underwent TKA in our hospital between January 1, 2017 and December 31, 2021 were offered preoperative lower extremity ultrasounds.

**Fig. 1:**
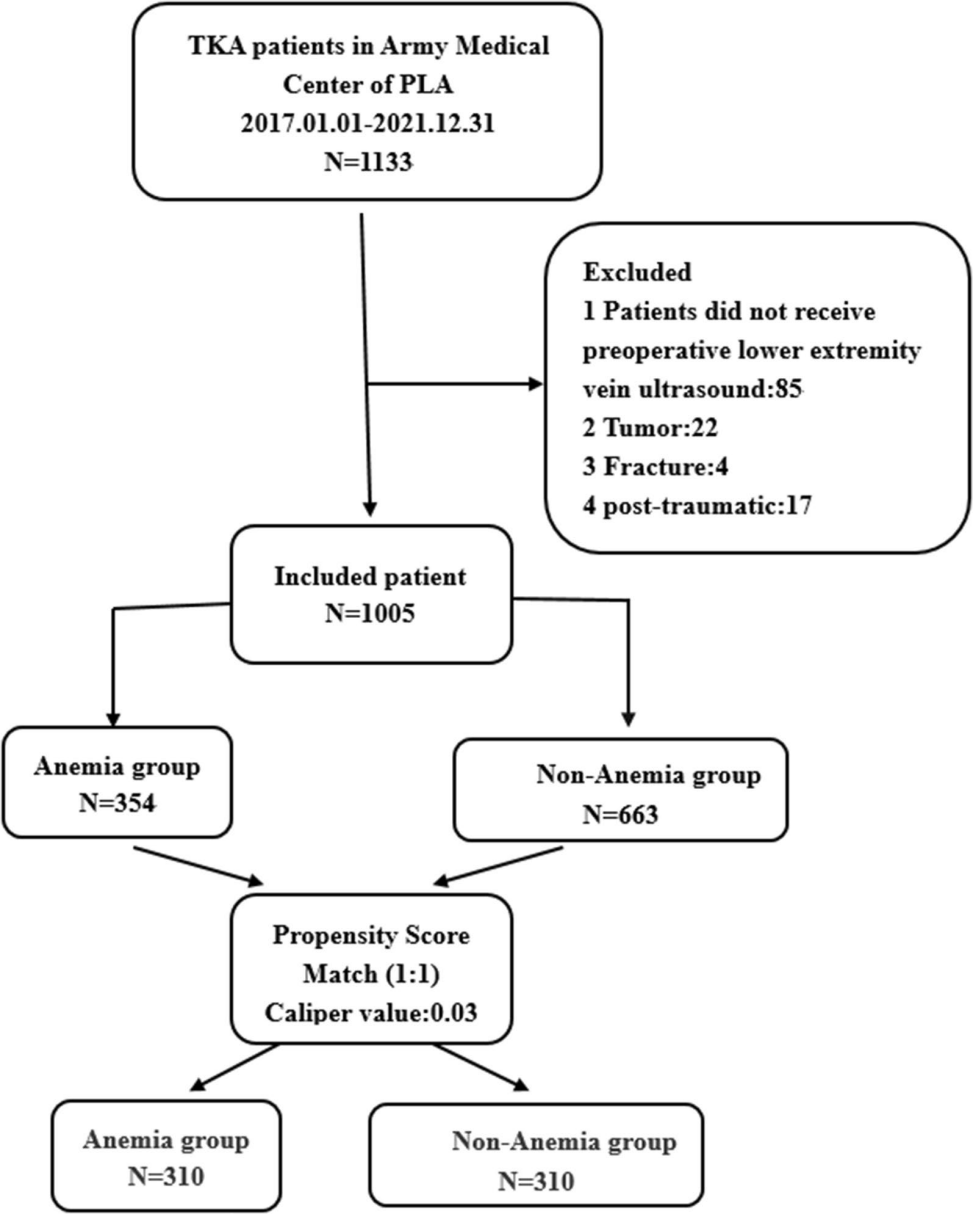
The research route of PSM in preoperative anemia and DVT in TKA patients. *TKA*—total knee arthroplasty; *DVT*—deep vein thrombosis; *BMI*—body mass index; *DM*—diabetes mellitus; *OA*—osteoarthritis; *CHD*—coronary heart disease; *COPD*—chronic obstructive pulmonary disease;

*Exclusion* Of 1133 patients, 1005 met entry criteria, excluding 85 with no lower extremity ultrasound, 22 with tumors, 4 with fractures, and 17 with trauma. (See Fig. 1).

## Study design

1005 patients with KOA were enrolled in. As per the 2011 World Health Organization (WHO) guidelines\*MERGEFORMAT [9] (and the indicators used by our laboratory), when Hb < 130 g/L in men and < 120 g/L in women, it is defined as anemia. According to their preoperative Hb levels, patients were divided into anemia group and the non-anemia group. According to the results of the lower extremity vein Doppler ultrasonography, the TKA patients were assigned into DVT group and non-DVT group for investigating the relation between preoperative anemia and preoperative DVT in KOA patients. This study has been approved by the Army Medical Center of PLA; ratification number is 2021(288) and it has completed the WHO international clinical trials registry (ChiCRT2100054844). As this was a retrospective study, the informed consent of the patients was waived by the ethics committee.

## Data collection

Patients’ general information included name, admission number, height, weight, BMI, age, gender, preoperative diagnosis. Their medical records included: preoperative hypertension, diabetes mellitus (DM), coronary heart disease (CHD), chronic obstructive pulmonary disease (COPD), chronic bronchitis, rheumatoid arthritis (RA), osteoarthritis (OA), cerebral infarction, cancer, renal failure, use of corticosteroids, preoperative smoking, alcohol consumption, major surgery within 12 months; laboratory examinations and auxiliary examinations: blood type (type A, B, AB, O), routine blood test (Hb, RBC count, hematocrit), the result of preoperative low extremity vein ultrasound.

All patients were examined by Philips IE33 GE Vivid 9, C5-1 and 5-10 Hz pulsed Doppler ultrasound. Each patient was co-diagnosed by two experienced sonographers. The positive criteria for DVT were venous incompressibility, intravascular filling defects, and lack of Doppler signal. We also collected the sites of DVT formation: distal, proximal and mixed thrombus formation. Distal thrombus (thrombus far from the popliteal vein); proximal thrombus (thrombus near the popliteal vein); mixed thrombus (thrombus contained both proximal and distal ends).

## Statistical analysis

Statistical analyses were performed using SPSS 26.0 software. Chi-Square test or Fisher’s Exact test was adopted for enumeration data. The results were represented in percentage (%) to analyze DVT-related covariates. To minimize the influence of confounders, a logistic model was established using PSM, taking preoperative anemia before TKA as the dependent variable and DVT-related variables as the covariates. Taking 0.03 as the Caliper value, anemia group and non-anemia group were matched at a 1:1 ratio. PSM matching results were evaluated using standard differences (d), which were calculated. If *d* < 0.1, it was evaluated as well-matched. After matching, binary logistic regression analyses were used to calculate the adjusted odds ratio (OR) and 95% Confidence Interval (CI), so as to evaluate the correlation of preoperative anemia before TKA and DVT. *P* value < 0.05 was considered statistically significant.

## Results

### General preoperative information of patients undergoing TKA

After inclusion and exclusion, among the 1133 patients who underwent TKA between January 1, 2017 and December 31, 2021, 1005 (88.7%) patients undergoing TKA met the criteria, with a sample loss rate below 20%. 208 cases (20.70%) were male and 797 cases (79.30%) were female. All basic information of patients is seen in Table 1. Anemia was defined as Hb < 130 g/L in men and < 120 g/L in women in patients undergoing TKA. 342 cases (33.7%) were defined as patients with anemia. 952 patients were preoperatively diagnosed with OA and 53 patients with RA. The preoperative comorbidities in TKA patients were hypertension (391 cases, 38.91%), DM (138 cases, 13.73%) and CHD (64 cases, 6.37%).

**Table 1:** Summary of patient characteristics

	Anemia(*N* = 342)	Non-Anemia(*N* = 663)	*P* value
Age (year)	69.87 ± 8.60	67.87 ± 8.2	0.000
Height (cm)	155.16 ± 6.85	155.99 ± 7.11	0.077
Weight (kg)	59.87 ± 9.39	62.84 ± 9.49	0.000
BMI(kg/m^2^)	24.87 ± 3.57	25.81 ± 2.46	0.000
Hb (g/L)	110.46 ± 10	133.03 ± 9.92	0.000
Hct (%)	34.38 ± 2.88	40.24 ± 2.5	0.000

*BMI*—body mass index; *Hb*—hemoglobin; *Hct*—hematocrit

*P* < 0.05 was considered statistically significant

## Comparison of DVT-related variables before and after PSM

Before PSM, the p value of blood type, hypertension, CHD, major surgery history in the last 12 months, chronic bronchitis, cerebral infarction, history of smoking > 0.05, and there was no statistic difference between anemia group and non-anemia group. While the two groups had statistic differences with *P* value < 0.05 in terms of gender (*P* = 0.000), age group (*P* = 0.01), DM (*P* = 0.032), type of knee arthritis (*P* = 0.001), alcohol consumption (*P* = 0.016), renal failure (*P* = 0.002), use of corticosteroids (*P* = 0.000).

After PSM, 310 pairs of data successfully matched with the 16 variables’ *P* values > 0.05, and there were no statistic differences between the two groups. This demonstrates the two sets of data are balanced and comparable (Table 2).

**Table 2:** Distribution characteristics of covariates in TKA patients before and after propensity score matching in Anemia group and Non-Anemia group

Covariates	Before matching				*χ*2	*P* Value	After matching				*χ*2	*P* Value
	Anemia		Non-Anemia				Anemia		Non-Anemia			
	(*N* = 342)		(*N* = 663)				(*N* = 310)		(*N* = 310)			
*Gender*												
Male	74	21.64%	134	20.21%	0.28	0.00	68	21.94%	76	24.52%	0.579	0.253
Female	268	78.36%	529	79.79%			242	78.06%	234	75.48%		
*Age group(y)*												
≤ 60	40	11.70%	113	17.04%	13.53	0.01	32	10.32%	30	9.68%	0.133	0.717
60–70	134	39.18%	301	45.40%			127	40.97%	125	40.32%		
> 70	168	49.12%	249	37.56%			151	48.71%	155	50.00%		
*BMI (kg/m*^*2*^*)*												
< 18.5	11	3.22%	4	0.60%	15.49	0.00	3	0.97%	8	2.58%	2.459	0.483
18.5–24.9	176	51.46%	309	46.61%			162	52.26%	156	50.32%		
25–29.9	125	36.55%	264	39.82%			117	37.74%	116	37.42%		
≥ 30	30	8.77%	86	12.97%			28	9.03%	30	9.68%		
*ABO blood type*												
type A	113	33.04%	228	34.39%	2.242	0.524	30	9.68%	32	10.32%	0.133	0.936
type B	78	22.81%	166	25.04%			125	40.32%	127	40.97%		
type AB	31	9.06%	45	6.79%			155	50.00%	151	48.71%		
type O	120	35.09%	224	33.79%								
*Hypertension*												
Yes	123	35.96%	268	40.42%	1.886	0.096	113	36.45%	119	38.39%	0.248	0.678
No	219	64.06%	395	59.58%			197	63.55%	191	61.61%		
*DM*												
Yes	37	10.82%	101	15.23%	3.71	0.032	38	12.26%	33	10.65%		
No	305	89.18%	562	84.77%			272	87.74%	277	89.35%		
*CHD*												
Yes	26	7.60%	38	5.73%	1.324	0.155	22	7.10%	26	8.39%	0.364	0.653
No	316	92.40%	625	94.27%			288	92.90%	284	91.61%		
*History of major surgery in the last 12 months*												
Yes	12	3.51%	19	2.87%	0.312	0.351	9	2.90%	8	2.58%	0.06	0.5
No	330	96.49%	644	97.13%			301	97.10%	302	97.42%		
*Chronic bronchitis*												
Yes	6	1.75%	7	1.06%	0.862	0.258	5	1.61%	5	1.61%	1	0.624
No	336	98.25%	656	98.94%			305	98.39%	305	98.39%		
*Cerebral infarction*												
Yes	11	3.22%	12	1.81%	1.996	0.118	9	2.90%	8	2.58%	0	1
No	331	96.78%	651	98.19%			301	97.10%	302	97.42%		
*Classification of OA*												
RA	37	10.82%	16	2.41%	31.909	0.001	16	5.16%	16	5.16%	0	1
OA	305	89.18%	647	97.59%			294	94.84%	294	94.84%		
*History of drinking*												
Yes	4	1.17%	24	3.62%	5.001	0.016	4	1.29%	6	1.94%	0.84	0.5
No	338	98.93%	639	96.38%			306	98.71%	304	98.06%		
*History of Smoking*												
Yes	13	3.80%	29	4.37%	0.185	0.402	11	3.55%	11	3.55%	0	1
No	329	96.20%	634	95.63%			299	96.45%	299	96.45%		
*Renal failure*												
Yes	6	1.75%	0	0.00%	11.701	0.002	0	0.00%	0	0.00%	*	*
No	336	98.25%	663	100%			310	0.00%	310	0.00%		
*Use of corticosteroids*												
Yes	15	4.59%	3	0.45%	19.85	0	2	0.65%	3	0.97%	0.2	0.5
No	327	95.61%	660	95.55%			308	99.35%	307	99.03%		
*History of cancer*												
Yes	3	0.88%	6	0.90%	0.002	0.633	3	0.97%	4	1.29%	0.144	1
No	339	99.12%	657	99.10%			307	99.03%	306	98.71%		
*COPD*												
Yes	4	1.17%	14	2.11%	1.138	0.21	22	7.10%	26	8.39%	0.36	0.65
No	338	98.83%	649	97.89%			288	92.90%	284	91.61%		

*BMI*—body mass index; *DM*—diabetes mellitus; *CHD*—coronary heart disease; *OA*—osteoarthritis; *COPD*—chronic obstructive pulmonary disease; *DVT*—deep vein thrombosis

*P* < 0.05 was considered statistically significant

## Equilibrium of the two covariables before and after matching

**Fig. 2:**
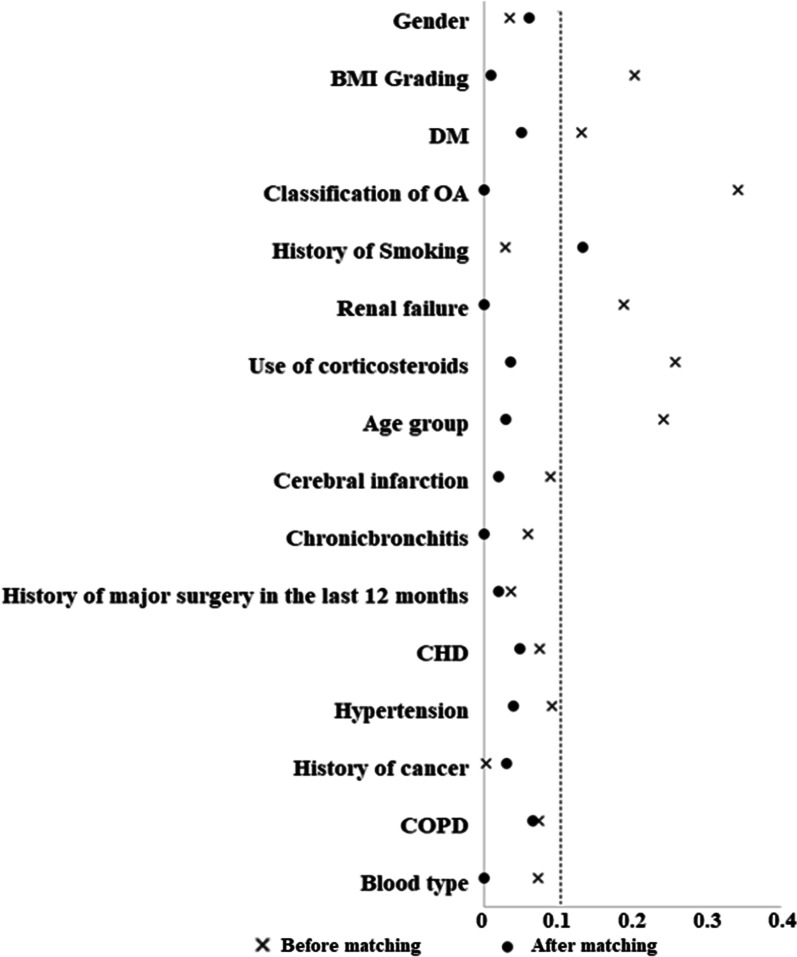
Variable standardization difference diagram. *OR*—odds ratio; *CI*—confidence interval; *PSM*—propensity score matching; *DVT*—deep vein thrombosis; *TKA*—total knee arthroplasty; *P* < 0.05 was statistically significant

Standardized differences (d) in the covariates of the two sets of data were calculated before and after matching. There were 16 covariates (gender, BMI grading, DM, classification of OA, history of smoking, renal failure, use of corticosteroids, age group, cerebral infarction, chronic bronchitis, history of major surgery in the last 12 months, CHD, Hypertension, History of cancer, COPD, Blood type) of data before PSM. Among them, the d values for six covariates (including BMI grading, age group, DM, etc.) of data were > 0.1. After PSM, excluding history of smoking (*d* = 0.133), 15 covariates of data all had d < 0.1, indicating that the PSM achieved good matching results (Fig. 2).

## Association between preoperative anemia and preoperative DVT in patients undergoing TKA

DVT occurred in 73 cases (7.2%) before TKA. We found 35 cases with DVT (10.23%) in the anemia group and 38 cases with DVT (5.73%) in the non-anemia group. After matching, there were 46 cases with DVT (7.42%) with 30 cases (9.68%) in the anemia group and 16 cases (5.16%) in the non-anemia group (Table 3). There were 52 cases (71.23%) with distal thrombus, 10 cases (13.7%) with proximal thrombus, and 11 cases (15.07%) with mixed thrombus. inferior vena cava filter was preoperatively implemented in all patients with proximal thrombi and mixed thrombi. Only one patient developed postoperative PE after TKA.

**Table 3:** Incidence of DVT in Anemia and Non-Anemia groups before and after PSM matching

	DVT			
	Before matching		After matching	
Non-Anemia	663(38)	5.73%	310 (16)	5.16%
Anemia	342(35)	10.23%	310 (30)	9.68%

**Fig. 3:**
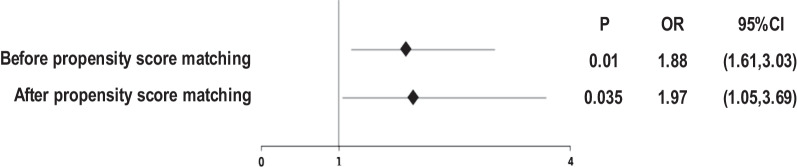
Binary logistic regression analysis of preoperative anemia and DVT in TKA patients

*DVT*—deep vein thrombosis; *PSM*—propensity score matching;

Before PSM, we found the risk for DVT in TKA patients with preoperative anemia increased by 1.88 times ([95% CI 1.16–3.03], *P* = 0.01). After PSM, the risk for DVT increased by 1.97 ([95% CI 1.05–3.69], *P* = 0.035) (Fig. 3).

## Discussion

Our study first found that after PSM, the risk for preoperative DVT formation in TKA patients with preoperative anemia increased by 1.97 times [95% (CI 1.05–3.69)], *P* = 0.035. Different from other studies, we investigated the association between preoperative anemia and DVT before surgery in terms of both inflammation and RBCs.

### Association between preoperative anemia and DVT in patients with KOA

Spahn DR. found that preoperative anemia was highly prevalent, ranging from 24 ± 9 to 44 ± 9% in patients undergoing THA, TKA and hip fracture surgery\*MERGEFORMAT [4]. In the present study, preoperative anemia occurred in 342 patients (33.7%), consistent with the incidence indicated by Spahn DR. *MERGEFORMAT [4]. Previous studies have found that 18–40% of patients undergoing TKA had received blood transfusion [10]. Low preoperative Hb level was considered a risk factor for moderate and severe postoperative anemia in patients with primary TKA [12]. Additionally, it has been found that preoperative anemia increased the risk for prosthetic-related infection in patients undergoing TJA [13]. In patient who underwent colorectal surgery, anemia is associated with the increased incidence of DVT and PE [14]. Xiong X and Bo Cheng found that the decrease in erythrocyte count was a high risk factor for preoperative DVT in KOA patients before TKA [15]. However, their study only revealed that the decrease in erythrocyte count contributed to preoperative DVT in TKA patients, and did not verify whether anemia is a high risk factor for preoperative DVT before TKA. Before and after PSM, we found the risk for DVT in TKA patients with preoperative anemia increased by 1.88 times ([95% CI 1.16–3.03], *P* = 0.01) and 1.97 times ([95% CI 1.05–3.69], *P* = 0.035), respectively.

### Possible mechanism of preoperative anemia and DVT in patients undergoing TKA

To date, anemia is defined by Hb levels in most studies. As per the WHO standards, the decrease in RBC counts (female < 3.5 × 10^12^/L, male < 4 × 10^12^/L) is also one of the indicators for diagnosing anemia\*MERGEFORMAT [9]. Both the inflammatory mechanism and the RBC mechanism could be plausible explanations of thrombosis formation in patients with preoperative anemia.

### Mechanism of inflammation

Over the last several decades, OA and the metabolic syndrome are increasingly recognized as the low-grade inflammatory condition with elevations in systemic inflammatory mediators such as Interleukin-1 (IL-1), Interleukin-6 (IL-6), and Tumor necrosis factor (TNF), and Interleukin-17 (IL-17)\*MERGEFORMAT [16]. In RA patients, IL-17 coordinates local inflammation, induces proinflammatory cytokines to prolong the inflammation process\*MERGEFORMAT [18]. In both OA and RA, IL-17 lead to cartilage inflammation [17]\*MERGEFORMAT [18]. During inflammation, IL-6 stimulates the liver to produce hepcidin that binds to ferroportin, decreasing intestinal iron absorption, the release of the iron stored in hepatocytes and macrophages, production of functional iron and RBC [20]. Cytokines, including TNF-α, IL-1, and IL-6, are produced after the onset of inflammation. These cytokines restrict RBC and shorten the lifespan of RBCs [21]. Elevation of C-reactive protein, IL-6, IL-8, and TNF during a response to systemic inflammation is associated with increased VTE risk [22]. Activation of endothelial cells, platelets, and leukocytes, onset of inflammation and formation of microparticles are the earliest events following thrombosis, which can trigger the coagulation system through the induction of tissue factor [22].

### Mechanism of RBC

Firstly, our data show that the number of platelets (PLTs) in anemia group patients are higher than that in non-anemia group patients. As the PLT count increases, more PLTs aggregate near the vessel wall. RBC deformability is mainly due to RBCs’ biconcave shape. The more rigid RBCs are, the more difficult it is for them to pass through capillaries, and the easier to marginate PLTs [23]. Together with the increase in PLT count, thrombosis can be easily formed. Symeonidis A and Athanassiou G found that DM, hypertension, VTE of the lower extremity, and CHD, could make RBCs very hard, likely leading to thrombosis [24]. And most of the patients in our study were aged patients with hypertension, CHD or DM. Secondly, during inflammation, RBCs were damaged [21]. In the view of membrane asymmetry of RBCs, phosphatidylserine were exposed in the context of cell injury induced by inflammation or oxidative stress [25]. Due to the large amount of RBCs in blood, even a small amount of them in contact with phosphatidylserine could lead to thrombosis [26]. Finally, RBCs may interact with the activated endothelial cells and/or expose and bind to subendothelial matrix, which leads to the formation of thrombosis [26].

### Optimization of preoperative anemia in patients undergoing TKA

It’s been proven that patient blood management implemented for patients undergoing TJA or TKA would decrease blood transfusion, length of stay, morbidity, and readmission [27]. Effective methods to preoperative correction of anemia include the use of erythropoietin (EPO) and oral or intravenous (IV) iron supplementation [28]. However, EPO may increase the risk of thromboembolism [29]. To ensure its safety and efficacy, EPO must be administered together with iron to enhance its therapeutic effect. In the meantime, measures to prevent thrombosis formation must be taken [30]. Oral iron is a low-cost way to treat anemia, but to be more effective, patients must take oral iron 3–6 month before surgery [28]. Compared with oral iron, intravenous iron therapy is faster in improving Hb levels and has better tolerance [31]. However, as intravenous iron administration is invasive and costs higher, Smith A and Moon, T do not recommend preoperative IV iron therapy for all patients scheduled for major orthopedic surgery [32].

The normal lifespan of human RBCs is approximately 120 days, and even if the lifespan is decreased by inflammation, a clinically significant reduction in erythrocyte count does not usually develop until weeks to months after the onset of the underlying inflammatory disorder [21]. The disease course of TKA patients is relative long, with a mean onset time ranging from months to decades, and most TKA patients are with advanced KOA, the surgeries for these patients are not life-threatening. Therefore, when preoperative anemia is found in TKA patients, they can choose the appropriate method to optimize the state of preoperative anemia.

## Strengths and limitations

In this study, the correlation between preoperative anemia and DVT in TKA patients was explored by using materials such as basic medical history, preoperative laboratory examinations, and preoperative auxiliary examinations. However, this study has certain limitations. As a retrospective study, some data are incomplete, and there existed the selection bias. The association between the severity of anemia and the formation of preoperative DVT will be further investigated.

## Conclusion

Preoperative anemia is an independent risk factor for preoperative DVT in KOA patients.

## Data Availability

The datasets used and/or analyzed during the current study are available from the corresponding author on reasonable request.

## Abbreviations

BMI: Body mass index
CHD: Coronary heart disease
COPD: Chronic obstructive pulmonary disease
CI: Confidence interval
DM: Diabetes mellitus
DVT: Deep vein thrombosis
EPO: Erythropoietin
HB: Hemoglobin
HCT: Hematocrit
IL-1: Interleukin-1
IL-17: Interleukin-17
IL-6: Interleukin-6
KOA: Knee osteoarthritis
OA: Osteoarthritis
OR: Odds ratio
PE: Pulmonary embolism
PSM: Propensity score matching
RA: Rheumatoid arthritis
RBC: Red blood cell
SF: Synovium fluid
TF: Tissue factor
THA: Total hip arthroplasty
TJA: Total joint arthroplasty
TKA: Total knee arthroplasty
TNF: Tumor necrosis factor
PLT: Platelets
VTE: Venous thromboembolism
WHO: World Health Organization

## References

[1] Goudie EB, Robinson C, Walmsley P, Brenkel I (2017). Changing trends in total knee replacement. Eur J Orthop Surg Traumatol Orthop Traumatol.

[2] Falck-Ytter Y, Francis CW, Johanson NA, Curley C, Dahl OE, Schulman S, Ortel TL, Pauker SG, Colwell CW (2012). Prevention of VTE in orthopedic surgery patients: antithrombotic therapy and prevention of thrombosis, 9th ed: American College of chest physicians evidence-based clinical practice guidelines. Chest.

[3] Bala A, Huddleston JI, Goodman SB, Maloney WJ, Amanatullah DF (2017). Venous thromboembolism prophylaxis after TKA: Aspirin, warfarin, enoxaparin, or factor Xa inhibitors?. Clin Orthop Relat Res.

[4] Spahn DR (2010). Anemia and patient blood management in hip and knee surgery: a systematic review of the literature. Anesthesiology.

[5] Khorana AA, Kuderer NM, Culakova E, Lyman GH, Francis CW (2008). Development and validation of a predictive model for chemotherapy-associated thrombosis. Blood.

[6] Coutinho JM, Zuurbier SM, Gaartman AE, Dikstaal AA, Stam J, Middeldorp S, Cannegieter SC (2015). Association between anemia and cerebral venous thrombosis: case–control study. Stroke.

[7] Potaczek DP, Jankowska EA, Wypasek E, Undas A (2016). Iron deficiency: a novel risk factor of recurrence in patients after unprovoked venous thromboembolism. Polskie Arch Medy Wewnetrznej.

[8] Cheung CL, Ang SB, Chadha M, Chow ES, Chung YS, Hew FL, Jaisamrarn U, Ng H, Takeuchi Y, Wu CH, Xia W, Yu J, Fujiwara S (2018). An updated hip fracture projection in Asia: the Asian Federation of Osteoporosis Societies study. Osteoporos Sarcopenia.

[9] World Health Organization. Haemoglobin concentrations for the diagnosis of anaemia and assessment of severity (No. WHO/NMH/NHD/MNM/11.1). World Health Organization (2011).

[10] Gombotz H, Rehak PH, Shander A, Hofmann A (2014). The second Austrian benchmark study for blood use in elective surgery: results and practice change. Transfusion.

[11] Hart A, Khalil JA, Carli A, Huk O, Zukor D, Antoniou J (2014). Blood transfusion in primary total hip and knee arthroplasty. Incidence, risk factors, and thirty-day complication rates. J Bone Joint Surg.

[12] Cao G, Yang X, Xu H, Yue C, Huang Z, Zhang S, Quan S, Yao J, Yang M, Pei F (2021). Association between preoperative hemoglobin and postoperative moderate and severe anemia among patients undergoing primary total knee arthroplasty: a single-center retrospective study. J Orthop Surg Res.

[13] Lu M, Sing DC, Kuo AC, Hansen EN (2017). Preoperative anemia independently predicts 30-day complications after aseptic and septic revision total joint arthroplasty. J Arthroplasty.

[14] Yeap E, Teoh W, Nguyen TC, Suhardja TS (2021). Preoperative anaemia and thrombocytopenia are associated with venous thromboembolism complications after colorectal resection. ANZ J Surg.

[15] Xiong X, Cheng B (2021). Preoperative risk factors for deep vein thrombosis in knee osteoarthritis patients undergoing total knee arthroplasty. J Orthop Sci.

[16] Huffman KM, Kraus WE (2012). Osteoarthritis and the metabolic syndrome: more evidence that the etiology of OA is different in men and women. Osteoarthr Cartil.

[17] Babaei M, Javadian Y, Narimani H, Ranaei M, Heidari B, Basereh H, Gholinia H, Firouzjahi A (2019). Correlation between systemic markers of inflammation and local synovitis in knee osteoarthritis. Casp J Intern Med.

[18] Kim KW, Kim HR, Kim BM, Cho ML, Lee SH (2015). Th17 cytokines regulate osteoclastogenesis in rheumatoid arthritis. Am J Pathol.

[19] Teerawattanapong N, Udomsinprasert W, Ngarmukos S, Tanavalee A, Honsawek S (2019). Blood leukocyte LINE-1 hypomethylation and oxidative stress in knee osteoarthritis. Heliyon.

[20] Lasocki S, Longrois D, Montravers P, Beaumont C (2011). Hepcidin and anemia of the critically ill patient: bench to bedside. Anesthesiology.

[21] Ganz T (2019). Anemia of inflammation. N Engl J Med.

[22] Branchford BR, Carpenter SL (2018). The role of inflammation in venous thromboembolism. Front Pediatr.

[23] Aarts PA, Banga JD, van Houwelingen HC, Heethaar RM, Sixma JJ (1986). Increased red blood cell deformability due to isoxsuprine administration decreases platelet adherence in a perfusion chamber: a double-blind cross-over study in patients with intermittent claudication. Blood.

[24] Symeonidis A, Athanassiou G, Psiroyannis A, Kyriazopoulou V, Kapatais-Zoumbos K, Missirlis Y, Zoumbos N (2001). Impairment of erythrocyte viscoelasticity is correlated with levels of glycosylated haemoglobin in diabetic patients. Clin Lab Haematol.

[25] Shi J, Shi Y, Waehrens LN, Rasmussen JT, Heegaard CW, Gilbert GE (2006). Lactadherin detects early phosphatidylserine exposure on immortalized leukemia cells undergoing programmed cell death. Cytom Part A J Int Soc Anal Cytol.

[26] Whelihan MF, Zachary V, Orfeo T, Mann KG (2012). Prothrombin activation in blood coagulation: the erythrocyte contribution to thrombin generation. Blood.

[27] Loftus TJ, Spratling L, Stone BA, Xiao L, Jacofsky DJ (2016). A patient blood management program in prosthetic joint arthroplasty decreases blood use and improves outcomes. J Arthroplasty.

[28] Lu Q, Peng H, Zhou GJ, Yin D (2018). Perioperative Blood management strategies for total knee arthroplasty. Orthop Surg.

[29] Lin DM, Lin ES, Tran MH (2013). Efficacy and safety of erythropoietin and intravenous iron in perioperative blood management: a systematic review. Transfus Med Rev.

[30] Muñoz M, Gómez-Ramírez S, Kozek-Langeneker S (2016). Pre-operative haematological assessment in patients scheduled for major surgery. Anaesthesia.

[31] Ng O, Keeler BD, Mishra A, Simpson JA, Neal K, Al-Hassi HO, Brookes MJ, Acheson AG (2019). Iron therapy for preoperative anaemia. Cochrane Database Syst Rev.

[32] Smith A, Moon T, Pak T, Park B, Urman RD (2020). Preoperative anemia treatment with intravenous iron in patients undergoing major orthopedic surgery: a systematic review. Geriatr Orthop Surg Rehabilit.

